# A Delphi study on valuing DNA sequencing in oncology: a European stakeholder developed framework for assessing next generation sequencing and comprehensive genomic profiling diagnostics

**DOI:** 10.1016/j.ebiom.2025.105947

**Published:** 2025-10-16

**Authors:** Augustovski Federico, Chavez Danitza, Haig Madeleine, Main Caitlin, Argento Fernando, Colaci Carla, Mills Mackenzie, Alfie Verónica, Pichon-Riviere Andrés, Alcaraz Andrea, Kanavos Panos

**Affiliations:** a Department of Health Technology Assessment and Health Economics, Institute for Clinical Effectiveness and Health Policy (IECS), Buenos Aires, Argentina; b School of Public Health, University of Buenos Aires School of Medicine (UBA), Buenos Aires, Argentina; c LSE Health, Medical Technology Research Group, London School of Economics (LSE), London, United Kingdom; d Department of Health Policy, London School of Economics (LSE), London, United Kingdom

**Keywords:** Value framework, NGS/CGP diagnostics, Health technology assessment, Precision medicine, Decision-making, Delphi panel, Next generation sequencing, Comprehensive genomic profiling

## Abstract

**Background:**

Advanced genomic technologies like Next Generation Sequencing and Comprehensive Genomic Profiling are pivotal for the prevention, management and treatment of cancer by identifying crucial genetic markers. However, their adoption in Europe is inconsistent, partly due to the lack of a validated approach to assessing their value.

**Methods:**

A multi-phase mixed-methods approach was implemented, integrating a systematic review and multi-stakeholder consensus-generating Delphi exercise to derive a comprehensive set of value criteria and arrive at a value assessment framework. This value assessment framework adapted an existing Latin American-focused diagnostic framework to the European context. The Delphi included representatives from the broader stakeholder community (patient advocacy, industry, decision-makers, health technology assessment, regulators, academia, and physicians). Over four rounds, participants refined and rated the significance of these criteria in the context of the assessment of the specified technologies in oncology, particularly for reimbursement decisions. Responses were analysed in terms of stability and level of consensus in order to generate a final value assessment framework.

**Findings:**

34 individuals participated in all rounds of the Delphi exercise. The final value assessment framework includes 8 distinct value criteria, including: clinical impact; test performance and quality; quality of scientific evidence; non-clinical impact; impact on health system integration, organisation and delivery of care; economic aspects; ethical and governance concerns; and health system priorities. Within these criteria, a total of 27 distinct sub-criteria were identified, 23 of which had consensus as ‘important’ or ‘very important’ in assessing value.

**Interpretation:**

The resultant value assessment framework is validated by a wide range of key European stakeholders and enables systematic assessment of Next Generation Sequencing and Comprehensive Genomic Profiling technologies used in oncology diagnostics within the European setting. The framework includes aspects that are not adequately considered in current health technology assessment and goes beyond existing value assessment frameworks through the inclusion of newer criteria such as data governance concerns.

**Funding:**

Funding was provided by the Precision Cancer Consortium with an unrestricted educational grant.

**Research in context**

**Evidence before this Study**

Evidence-informed decision-making is increasingly important to optimise access to innovative technologies in oncology as well as in the broader healthcare ecosystem. Value frameworks are crucial for determining key factors in guiding decision-making. With the ongoing advancements in precision or personalised medicine, notably through Next Generation Sequencing (NGS) and Comprehensive Genomic Profiling (CGP), there is a pressing need to have tools to assess their value. A recently published systematic review synthesised 43 value frameworks of health technologies and contributed to a collaborative value framework building process in oncology in Europe. Considering all criteria and sub-criteria, a total of 18 criteria and 36 individual sub-criteria were identified, which served for performing the collaborative Delphi exercise.

**Added value of this study**

In this multi-stakeholder consensus-building co-creative Delphi exercise across key stakeholders in Europe, we propose a NGS and CGP value framework specifically targeted for oncology. The proposed value criteria extend beyond conventional frameworks by incorporating aspects of performance, evidence, ethics, and governance.

**Implications of all the available evidence**

Our research provides a more thorough evaluation of the value of latest-generation genomic tests for European decision-makers, enhancing the understanding of the benefits and potential coverage implications. Furthermore, this work could also be useful for developing broader, updated, and more encompassing value frameworks that could be applied to other regions worldwide as well as in different healthcare fields.

## Introduction

Precision medicine is transforming cancer management by tailoring therapies to the genetic variability of tumours.^1^^,^^2^ The identification of patients with actionable genomic alterations, crucial for selecting targeted therapies, has shown enhanced response rates compared to conventional chemotherapy.^3^, ^4^, ^5^, ^6^ Next Generation Sequencing (NGS) and Comprehensive Genomic Profiling (CGP), as forms of massive parallel sequencing, represent a significant advancement in obtaining patients’ genomic information.^7^ The potential benefit of this approach is very attractive to patients for whom no conventional therapy is available and for whom a CGP could identify a possible new and targeted therapeutic approach. Of these, NGS is one of the most advanced technologies applied to decipher molecular alterations in tumours.^7^ These technologies are key for tailored treatments and reducing wasteful healthcare spending.^1^^,^^8^ Yet, the potential of precision medicine remains unfulfilled if patients lack access to the necessary biomarker testing essential for determining their eligibility for treatments. To homogenously integrate NGS/CGP diagnostics into European healthcare and improve equitable access, stakeholder-informed holistic Value Assessment Frameworks (VAFs) are urgently needed.

Integrating NGS/CGP into clinical cancer care presents several challenges, including the complexity of interpreting genetic variants and distinguishing actionable mutations from benign ones. The application of clinical tiering systems is also difficult due to variations in evidence requirements across guidelines. Additionally, issues such as long turnaround times and concerns about treatment toxicity further complicate its routine use in clinical practice.^9^

Despite NGS endorsement by the European Society for Medical Oncology (ESMO) and its inclusion within the EU Beating Cancer Plan, less than 10% of specimens requiring molecular testing are analysed with NGS, with many countries testing fewer than 2% of tumours, highlighting the slow and uneven adoption across Europe due to disparities in healthcare policies, reimbursement rates, and levels of regional support.^2^^,^^10^^,^^11^ Funding complexities and a lack of clear value assessment contribute to access disparities and affordability issues. Recognising the need for VAFs is crucial as NGS/CGP technologies become central to enhancing population health, elevating the quality of care, and improving efficiency in resource allocation.^8^^,^^12^

Traditional Health Technology Assessment (HTA) methods fall short for evaluating advanced diagnostics like NGS/CGP.^12^^,^^13^ Assessing the value of these diagnostics is complex, given their role in informing treatment decisions and the inherent challenges involved in attributing value to individual components of care pathways.^2^ In particular, modelling the cost-effectiveness of NGS/CGP tests, linked to subsequent treatments, adds to the complexity of value assessments. They also face unique regulatory and reimbursement challenges due to their evolving nature post-approval, requiring adaptable assessment methods. Therefore, assessing the value of NGS and CGP requires comprehensive methodologies to cover all outcomes and total costs of the expected healthcare pathway, often over a patient's lifetime, and remain flexible enough to incorporate continuous technological advancements.^12^^,^^14^^,^^15^

Current VAFs for genetic testing, like those in the US and the UK, do not fully address the intricacies of technologies such as NGS/CGP.^12^ They often miss broader issues pivotal to precision medicine or are adopted inconsistently. Other initiatives, including the Evaluation of Genomic Applications in Practice and Prevention, overlook the nuanced challenges of genomic panels.^16^ Criteria such as analytical validity and penetrance require explicit consideration.^17^^,^^18^ Additionally, the ethical and legal aspects (e.g., incidental genomic findings), necessitate careful deliberation.^8^^,^^19^, ^20^, ^21^ While current frameworks give more weight to these ethical concerns, they do not fully capture the comprehensive value of NGS technologies. There is a clear need for healthcare systems to update existing frameworks to embrace the full value spectrum of these advanced diagnostic tools.

A holistic framework for NGS/CGP is crucial to recognising, measuring, and fully leveraging the benefits of diagnostics for patients, health systems, and society. This collaborative study between the London School of Economics (LSE) and the Institute for Clinical Effectiveness and Health Policy (IECS) aims to adapt the IECS VAF for diagnostics in Latin America. This earlier endeavour performed a systematic review, developed and piloted a collaborative framework for diagnostic technologies in the Latin American region.^18^^,^^22^ The current adaptation to NGS/CGP, rooted in a web-Delphi methodology and a systematic review for comprehensively identifying potential value dimensions,^18^ seeks to provide a comprehensive value framework reflecting European stakeholder values for NGS/CGP diagnostics in oncology.

## Methods

The study utilised a mixed-methods approach, including a systematic review and web-Delphi exercise to validate and generate consensus on value dimensions identified in the literature. The research was split into the following stages: 1) identification of value criteria, 2) stakeholder recruitment, 3) web-Delphi qualitative analysis and 4) quantitative analysis and framework validation (Table 1). A detailed description of the research methodology and the study protocol are provided in Supplemental Material.

**Table 1:** Study methodology.

	Components	Objectives	Methods	Tasks
First stage	Steering committee	Appoint a steering committee	Qualitative	Identify and invite potential steering committee members
	Systematic literature review	Generate initial framework for use in Delphi exercise	Qualitative	Conduct a systematic review to understand key value concerns and adapt broad value assessment framework (VAF) focussed on diagnostic technologies in the Latin American context to be focussed on Next Generation Sequencing and Comprehensive Genomic Profiling within the European context
	Initial list of criteria and sub-criteria	Create an initial VAF	Qualitative	Generate initial list of criteria and sub-criteria from systematic review results
Second stage	Stakeholder identification	Identify and invite stakeholders	Qualitative	Identify and invite stakeholders from existing networks
Third stage	Delphi exercise R1	Participants asked for qualitative feedback on proposed VAF	Qualitative	In R1 stakeholders are presented with the initial VAF and are asked to comment on existing sub-criteria and propose their own.
	Thematic analysis	Thematically analyse participant contributions in R1	Qualitative	Thematic analysis was completed using Excel to identify themes within participant comments and proposed sub-criteria
	Update VAF	Update initial proposed VAF with stakeholder proposed themes	Qualitative	Initial VAF updated to include all participant proposed themes that were not present in initial version.
Fourth stage	Delphi exercise R2 and R3	Consensus building exercise to understand where there is consensus	Quantitative	Delphi exercise R2—stakeholders asked to rate each criteria and sub-criteria on an ‘importance’ Likert scale. Delphi exercise R3—stakeholders shown how their response differed from the groups in R2 and are asked to re-rate criteria and sub-criteria
	Stability analysis	Analysis of participant response stability	Quantitative	Difference in participant responses between R2 and R3 analysed using the non-parametric Kruskall Wallis test
	Delphi exercise R4	Unstable criteria and sub-criteria retested	Quantitative	Delphi exercise R4—stakeholders asked to rate unstable criteria and sub-criteria again
	Data analysis	Analysis of respondent data within and between groups to determine respondent stability and consensus	Quantitative	Likert scale data analysed for each criteria and sub-criteria. Interquartile range, median, and stability calculated for each criteria and sub-criteria.

Abbreviation notes. DHT: Digital health technologies. VAFs: Value Assessment Frameworks. LSE: London School of Economics. HCPs: Health care professionals. R1, R2, R3: Round 1, Round 2 and Round 3 of the Delphi process.

A steering committee oversaw and provided guidance on the study. The committee included seven European stakeholders with expertise across patient advocacy, genomics, pathology, HTA and market access. Please see acknowledgements for steering committee names.

### Ethics

Ethics approval was received from the London School of Economics ahead of the Delphi exercise (Ref: 188696). Funding was received through an unrestricted educational grant from the Precision Cancer Consortium. The study adhered to the Accurate Consensus Reporting Document (ACCORD) guideline for reporting consensus-based methods.^23^ All participants were given a participant information sheet and signed a consent form via email. Participation in the Delphi was anonymous and the evidence collected represents the views of the individual participants, not their affiliations. Participants who completed four Delphi rounds were compensated €500 each by the LSE.

### Stage one: identification of value criteria

A systematic literature review (SLR) was conducted to update IECS's Latin American (LATAM)-focussed diagnostic VAF^17^ to focus on NGS/CGP oncology diagnostics in Europe.^5^ Eighteen value criteria and 36 sub-criteria were identified through the SLR.^18^ The SLR protocol and results are reported in full in Value in Health.^24^

### Stage two: stakeholder recruitment

Participants were identified from the London School of Economics (LSE) network of affiliated institutions and policy experts, which includes the ADVANCE HTA consortium, the IMPACT-HTA consortium, the World Health Organisation (WHO) Europe Collaborating Centres, health insurance/payer organisations, HTA agencies, regulatory agencies, professional organisations (e.g. the European Society for Medical Oncology – ESMO), and patient advocacy groups, among others. Participants were grouped into key stakeholder groups consisting of patient advocates, industry, decision-makers (those making funding decisions), HTA agencies, regulators, academia, and physicians. Generally, Delphi exercises range from under ten to several hundred participants^25^; given the specialised nature of genomic testing, we aimed to recruit ten participants per stakeholder group. Individuals with direct ties to the study sponsor were excluded from participation.

### Stage three: Web-Delphi qualitative analysis

The Delphi method is a scientific approach designed to organise expert discussions, systematically facilitating insights into controversial subjects with scarce information.^26^^,^^27^ This study employs the Delphi technique to systematically gather expert opinions on the value preferences of NGS/CGP technologies, an area with limited published research. The approach includes several ‘rounds’ of feedback, including an initial open-ended response round and a number of scoring rounds utilising a Likert scale.^26^^,^^28^ The initial qualitative round was used to collect stakeholder opinions, while the subsequent quantitative rounds generate consensus across stakeholders, validating these opinions to generate a co-created framework. For this study, the Delphi was conducted online, using the Welphi platform.^29^

Four rounds of the Delphi method were carried out: in round one (R1), participants shared their thoughts on the suggested criteria and sub-criteria from the initial framework and could introduce their own; in round two (R2), they evaluated each sub-criteria using a 5-point Likert scale for “importance” (ranging from “not at all important” to “very important”); in round three (R3), participants received feedback on the response distribution for each criteria and sub-criteria, including their own, and had the opportunity to revise their responses; finally, round four (R4) involved analysing the stability of criteria and sub-criteria between rounds two and three, retesting those deemed unstable with participants.

#### Delphi round one

Round one (R1) ran from 21st to 31st March 2023. Participants were provided with the list of initial criteria and sub-criteria identified from the SLR^24^ and were asked to add potential missing criteria/sub-criteria and comment on the inclusiveness, appropriateness, and clarity of each criteria/sub-criteria proposed.

#### Framework adaptation

A thematic analysis of the participant comments and proposed sub-criteria was conducted using Microsoft Excel. Two researchers independently thematically analysed the participant comments and proposed sub-criteria, labelled them according to core themes and sub-themes, and recommended modifications to the framework presented in R1. These independent analyses were then compared to each other and a consensus meeting including all authors was organised. In this meeting, authors aligned on the proposed framework modifications and ensured all themes identified from participant feedback in R1 were incorporated in the resultant framework. Criteria and sub-criteria were redefined, renamed, merged or deleted altogether according to this process.

### Stage four: quantitative analysis and framework validation

#### Delphi rounds two–four

Rounds two-four (R2-R4) ran from May to July 2023. In R2, participants were shown the value framework that was adapted based on the thematic analysis of R1 feedback. Participants then scored each criteria and sub-criteria on a 5-point Likert scale, according to their importance within a framework used as a decision aid for reimbursement and coverage decisions of NGS/CGP diagnostics. In R3, participants were shown how their responses differed from the overall cohort's responses and could change their answers or keep them the same. Individual participant responses were analysed for stability using the non-parametric Wilcoxon's test.^26^^,^^28^ In R4, criteria and sub-criteria with stable responses were excluded from scoring and those with unstable responses were scored a final time.

#### Statistics

Several statistical tests were completed using STATA 16.1 software^30^ to determine consensus, stability and descriptive statistics such as median responses across the cohort. Consensus was calculated using the interquartile range (IQR), where we defined consensus as a criteria having an IQR of ≤1. Wilcoxon's test was used to calculate the stability of responses between R2 and R3 and between R3 and R4 to understand if participants were actively changing their minds.

Inclusion criteria for the final framework considered both consensus (IQR ≤1) and the median importance rating of each sub-criteria. A median of ‘important’ and ‘very important’ resulted in the sub-criteria being considered ‘essential’ for value assessment, and median of ‘moderately important’ or lower resulted in the sub-criteria being considered as ‘complementary’ for assessment.

### Role of funders

The sponsor had no role in study design, data collection, data analyses, interpretation, or writing of the research.

## Results

The generation of this co-created value framework started with an initial set of 18 criteria and 36 sub-criteria, as identified by an SLR.^18^ This was adapted to 8 criteria and 29 sub-criteria, through participant qualitative feedback in R1 (see Supplemental Table S1 for a full list of value criteria and definitions). Following the validation process of web-Delphi R2-R4, the framework resulted in 23 ‘essential’ sub-criteria and four ‘complementary’ sub-criteria. Two sub-criteria were excluded due to not satisfying the inclusion criteria.

### Web-Delphi panel participants

Eighty-one European participants were invited to participate and, of those invited, 43 accepted and 34 completed R4, giving a 79% retention rate. The participants were from 12 European countries (Austria, Belgium, Denmark, France, Germany, Italy, Slovenia, Spain, Sweden, Switzerland, Netherlands, United Kingdom) and were classified into the following stakeholder groups: HTA (n = 5), regulatory bodies (n = 3), academia (n = 8), patient advocates (n = 6), physicians (n = 4), industry (n = 4), and decision-makers (n = 4) (Supplemental Table S2).

### Sub-criteria alteration from R1 thematic analysis

R1 resulted in 689 participant comments and 29 proposed sub-criteria, which were analysed by three independent researchers. R1 feedback and the thematic analysis guided the reformulation of the VAF. The analysis led to the identification of 34 key themes that informed the updated VAF in R2. Of the initial 46 sub-criteria, 14 sub-criteria were adapted, 22 descriptions were adapted, 11 sub-criteria were merged, 14 were deleted and 1 was kept the same. Moreover, the thematic analysis led to restructuring the broad criteria by clustering related themes into distinct categories; see Supplemental Table S3 for changes made to proposed sub-criteria.

### Consensus and stability measurements

All broad criteria reached consensus in R4 with IQRs of ≤1 (Table 2). Furthermore, the median participant responses were all either ‘important’ or ‘very important’ in R4. The criteria ‘clinical impact’, ‘test performance and quality’, and ‘quality of scientific evidence’ had median ratings of ‘very important’.

**Table 2:** Value Criteria Delphi results.

Value criteria	Description	IQR (25th–75th percentile)	Median
Clinical impact	The effect or influence that a diagnostic test has on patients' health outcomes, including clinical effectiveness, safety and the consequences of a wrong diagnosis.	0 (5–5)	Very important
Test performance and quality	The test's capacity to accurately identify or detect a specific condition or parameter of interest (i.e. sensitivity, specificity), which also encompasses the technical and quality assurance aspects of the test.	1 (4–5)	Very important
Quality of scientific evidence	The validity, credibility, and overall strength of the evidence used to support the decision to use NGS/CGP.	1 (4–5)	Very important
Non-clinical impact	The broader effects of NGS/CGP, extending beyond clinical outcomes. It encompasses the environmental impact of genetic testing practices, the implications for patients, caregivers and families, and their experiences during the testing and treatment process. Additionally, it considers how NGS/CGP influences personal or family decisions.	0 (4–4)	Important
Impact on health system integration, organisation and delivery of care	Effects of NGS/CGP on the healthcare system, including its implementation, influence on healthcare service provision, and appropriateness of test use.	1 (4–5)	Important
Economic aspects	Value-for-money, affordability, the financial impact on patients and families and the broader socioeconomic impacts of NGS/CGP testing.	1 (4–5)	Important
Ethical and governance concerns	Ethical, legal, and data governance concerns of NGS/CGP.	0 (4–4)	Important
Health system priorities	Alignment of NGS/CGP with the broader priorities of the health system, including disease burden and severity, meeting unmet needs and improving health equity.	0 (4–4)	Important

All criteria had stable participant responses and had a median between ‘important’ or ‘very important’. Note: ‘impact on health system integration, organisation and delivery of care’ had a median of 4.5 (i.e. between ‘important’ and ‘very important’, which was conservatively rounded down to ‘important’.

Consensus is measured as IQR <2, thus all criteria had consensus surrounding them. For the 25% and 75% percentile values, the numerical likert scale is shown. In the scale 1 = not at all important, 2 = little importance, 3 = moderately important, 4 = important, 5 = very important.

Of the sub-criteria only two had an IQR = 2, lacking consensus. These were ‘cancer stigma’ and ‘appropriateness of test use’. Thirteen sub-criteria were unstable between R2 and R3, and thus were tested further in R4 (Table 3). Of the 13 unstable sub-criteria, six remained unstable after R4. Regarding the median responses, most sub-criteria had a median rating of ‘important’, including ‘test safety’, ‘patient experience’, ‘bridge to other future treatments’, or ‘very important’, including ‘clinical efficacy’, ‘cost-effectiveness’ and ‘data security and privacy’ (Table 3). Four sub-criteria had a median response of ‘moderately important’, including ‘caregiver and/or family experience’ and ‘environmental impact’, ‘broader socioeconomic impact’ and ‘research priorities’.

**Table 3:** Sub-criteria stability and consensus.

Value criteria	Sub-criteria	Wilcoxon test Z values R2-R3 (P values)	Wilcoxon test Z values R3-R4 (P values)	IQR (25th–75th percentile)	Median
Clinical impact	Clinical efficacy/effectiveness	1.414 (0.157)		**0 (5–5)**	**Very important**
	Test safety	0.000 (1.00)		**1 (4–5)**	**Important**
	Consequences of wrong diagnosis	2.826 (0.005)∗	−1.000 (0.317)	**0 (5–5)**	**Very important**
Test performance and quality	Test performance	2.828 (0.005)∗∗	−2.000 (0.0455)∗	**0 (5–5)**	**Very important**
	Technical aspects	2.997 (0.003)∗∗	−2.236 (0.0253)∗	**1 (4–5)**	**Very important**
Quality of scientific evidence	Quality of scientific evidence	2.449 (0.0143)∗	−2.449 (0.014)∗	**1 (4–5)**	**Very important**
Non-clinical impact	Environmental impact	−1.342 (0.180)		*1 (3–4)*	*Moderately important*
	Patient experience	0.577 (0.564)		**0 (4–4)**	**Important**
	Caregiver and/or family experience	−0.447 (0.6547)		*1 (3–4)*	*Moderately important*
	Impact on personal and family decisions	2.642 (0.008)∗	0.000 (1.000)	**1 (4–5)**	**Important**
	Cancer stigma	−0.447 (0.655)		***2(2–4)***	***Important***
	Bridge to other future treatments (¨Real option value¨)	1.732 (0.083)		**0 (4–4)**	**Important**
Impact on health system integration, organisation and delivery of care	Impact on health service provision	3.158 (0.002)∗∗	−2.449 (0.014)∗	**1 (4–5)**	**Very important**
	Appropriateness of test use	1.732 (0.083)		***2(3–5)***	***Important***
Economic aspects	Cost-effectiveness	2.644 (0.008)∗∗	−2.121 (0.034)∗	**1 (4–5)**	**Very important**
	Affordability	2.644 (0.008)∗∗	−1.000 (0.317)	**1 (4–5)**	**Very important**
	Financial impact on patients, carers or family	0.031 (0.976)		**1 (3–4)**	**Important**
	Broader socioeconomic impact	−1.000 (0.317)		*1 (3–4)*	*Moderately important*
Ethical and governance concerns	Data security and privacy	3.000 (0.003)∗∗	−1.342 (0.180)	**1 (4–5)**	**Very important**
	Informed consent and transparent communication	2.236 (0.025)∗	−2.000 (0.046)∗	**1 (4–5)**	**Very important**
	Data provenance	0.447 (0.655)		**0 (4–4)**	**Important**
	Ethical aspects	1.732 (0.083)		**1 (3–4)**	**Important**
	Legal aspects	0.000 (1.000)		**1 (3–4)**	**Important**
Health system priorities	Disease burden	1.032 (0.302)		**0 (4–4)**	**Important**
	Disease severity	2.448 (0.014)∗	−2.236 (0.025)∗	**1 (4–5)**	**Very important**
	Unmet need	2.000 (0.046)∗	−1.342 (0.180)	**1 (4–5)**	**Very important**
	Research priorities	−1.369 (0.171)		*1 (3–4)*	*Moderately important/important*
	Equity	2.644 (0.008)∗∗	−1.890 (0.059)	**1 (4–5)**	**Very important**
	Public and population health	−0.816 (0.414)		**0 (4–4)**	**Important**

All proposed sub-criteria in rounds two—four are showcased. Wilcoxon test analyses whether there is a statistically significant difference in the median values between the rounds. Sample sizes range between 3 and 8. For the 25% and 75% percentile values, the numerical likert scale is shown. In the scale 1 = not at all important, 2 = little importance, 3 = moderately important, 4 = important, 5 = very important.

Asterisk showcases statistical significance, with ∗P < 0.05, ∗∗P < 0.01.

**Essential sub-criteria**.

*Complementary sub-criteria*.

***Sub-criteria with no consensus***.

Clinical efficacy was the only sub-criteria with an IQR = 0 and a median rating of ‘very important’, indicating clear consensus of its inclusion within the framework.

### Final value assessment framework

The final VAF resulted in 27 total sub-criteria grouped within 8 criteria, with 23 classed as ‘essential’ and 4 classed as ‘complementary’ (Table 4).

**Table 4:** Final framework.

Value criteria	Sub-criteria
**Essential sub-criteria**	
Clinical impact	Clinical efficacy/effectiveness
	Test safety
	Consequences of wrong diagnosis
Test performance and quality	Test performance
	Technical aspects
Quality of scientific evidence	Quality of scientific evidence
Non-clinical impact	Patient experience
	Caregiver and/or family experience
	Impact on personal and family decisions
	Bridge to other future treatments (¨Real option value¨)
Impact on health system integration, organisation and delivery of care	Impact on health service provision
Economic aspects	Cost-effectiveness
	Affordability
	Financial impact on patients, carers or family
Ethical and governance concerns	Data security and privacy
	Informed consent and transparent communication
	Data provenance
	Ethical aspects
	Legal aspects
Health system priorities	Disease burden
	Disease severity
	Unmet need
	Equity
	Public and population health
**Complementary sub-criteria**	
Non-clinical impact	Environmental impact
	Caregiver and/or family experience
Economic aspects	Broader socioeconomic impact
Health system priorities	Research priorities

Essential sub-criteria—median response of ‘important’ or ‘very important’ with consensus (IQR ≤1).

## Discussion

Current HTA methods do not accurately value DNA sequencing diagnostics in oncology. Our research -informed by the best available scientific evidence- explored the value preferences of a variety of multidimensional healthcare stakeholders in Europe in regards to NGS/CGP technologies used within oncology. Strong consensus was found across a variety of criteria, which has resulted in a novel co-created value framework.

The final framework, similar to other VAFs in this area, includes core assessment criteria covering clinical and economic impact, impact on system organisation and care delivery, and priority within the health system.^5^ However, a major differentiating factor of this framework is that it includes test performance and quality, quality of scientific evidence, and ethical and governance concerns.^5^ By extension, the sub-criteria identified with the highest importance ratings included several expected categories such as ‘clinical efficacy’, ‘affordability’, ‘unmet need’, and ‘equity’, but also several sub-criteria such as ‘data security and privacy’ and ‘impact on health service provision’ which have been traditionally missing or under-represented in existing VAFs.

The Data Governance criteria, in particular, were a standout issue for consideration in NGS/CGP value assessment that current HTA methods and policies typically overlook. Indeed, based on the IECS SLR, there is no other diagnostic VAF that considers data privacy and governance concerns after the test is performed.^18^ Health systems utilise HTA not only to understand cost-effectiveness and promote an efficient allocation of resources, but also to add transparency and accountability into decision-making processes. This is critical for establishing legitimacy and trust healthcare payers, but also in communicating clear value signals reflective of societal preferences to developers of health technologies. Omission of data governance considerations is a crucial misstep towards accurate value assessment because overlooking data management procedures neglects an entire aspect of the value chain. Stakeholders expressed clear concerns over the impact of widespread genomic profiling without reliable and robust data infrastructure that guarantees individual data rights of privacy and security. HTA needs to reflect real societal values and health system utilisation to be accurate.

Both the results from the thematic analysis (round 1 of the Delphi) and the final VAF highlight the importance of value criteria for NGS and CGP technologies that extend beyond clinical- and cost-effectiveness. Numerous stakeholders routinely expressed concerns related to non-clinical impact, health system integration, ethics, data governance access inequity, among others. For example, participants commented on health system integration and data governance stating, “Consider how this equipment will inter-operate with information systems within hospitals” and “The immutability of the data should be built into the technology... Ethical standards should not be an after-thought but made clear to the individual patient from the very start, not as an additional policy document to be provided or signed but as a technological default available to all.”

Current HTA frameworks, primarily those that are focused on the incremental cost-effectiveness ratio (ICER), encounter limitations when applied to NGS/CGP technologies, which provide several dimensions of benefits that extend beyond clinical and cost-effectiveness. When considering potential HTA methodological improvements to assess diagnostics, it is important to acknowledge the role NGS/CGP diagnostics will play in health systems moving forward, as NGS/CGP diagnostics can rarely be evaluated in isolation. As the precision oncology sector continues to grow and budgets for cancer care continue to have separate funding decision processes from other new pharmaceuticals, NGS/CGP diagnostics will only expand in use throughout the cancer care delivery pathway, as well as clinical research development pathways for new cell and gene therapies. For example, NGS/CGP diagnostics will increase the use and development of biomarker-driven therapies and the development of targeted molecular agents for identified genomic alterations within tumours.^31^ The critical dependence of diagnostic technologies on the benefits derived from associated therapies, coupled with the constant development of new therapies, poses challenges for ensuring a comprehensive, long-term assessment.

This challenge is well-illustrated in the context of current HTA frameworks that adopt co-dependent assessment of drugs and diagnostics, primarily comparing costs and benefits related to using diagnostic tests for targeted treatment compared to no testing or the use of standard treatment. It is unclear if this approach is fit-for-purpose for NGS/CGP, as these diagnostic technologies can be linked to numerous and continually expanding treatments. Moreover, NGS/CGP diagnostics are integral tools used throughout the continuum of care beyond initial diagnosis and treatment selection to ongoing monitoring, resistance detection, therapy adaptation and long-term disease management.^32^ Their role as a companion diagnostic persists throughout the treatment lifecycle, even informing clinical trials, by providing extensive genetic information crucial for the selection, monitoring, and adaptation of targeted therapies in cancer treatment.^32^

The consideration of both “direct” and “indirect” value criteria and sub-criteria represents a strength of this framework and provides for a more complete assessment of the value of NGS/CGP technologies. The former comprises health benefits derived from treatment decisions based on test results, which can be captured in new therapy trials, including clinical outcomes and health-related quality of life. However, indirect benefits that arise from the “value of knowing” and personal utilities are not routinely incorporated into clinical trial instruments, limiting the assessment of these diagnostic techniques under current HTAs, where the primary data sources are clinical trials and real-world data.^14^^,^^15^ In the present context, it is crucial to identify and quantify the real value of these technologies which yield multiple distinct outcomes. Such incentive and regulatory alignment efforts will optimise resource allocation and empower sustainable financing of high-cost genomic technologies such as NGS/CGP diagnostics.

Robust data infrastructure is needed to facilitate widespread use of NGS/CGP diagnostics and the resultant data in a secure and privacy-preserving way. It is well known that there are multifaceted challenges regarding NGS/CGP data infrastructure, including data quality and structuring, interoperability and clinical workflow integration, and standardisation of reporting.^33^ Oncologists are increasingly confronted with the challenge of incorporating a vast, changing and expanding body of genomic knowledge into patient care without advanced data infrastructure to support them.^33^ This task is further complicated by the growing prevalence of direct-to-consumer genomic profiling products, which may introduce data that do not align with standard clinical protocols yet must be integrated and addressed within the healthcare setting, often by general practitioners who are not trained in genetic counselling.^34^ There are also several social and ethical concerns to consider, including disclosure of incidental findings and overflow effects of diagnostic information onto family members. These interdisciplinary issues that straddle the boundaries of health systems illustrate some of the ways that NGS/CGP diagnostics create challenges in conducting HTAs that accurately assess their value and health system impact. Implementation of robust data infrastructure will help improve the quality of care, equity and access in personalised medicine through clinical standardisation resulting in uniform availability, cross-country data integration, and variant reporting.^35^ The lack of other VAFs considering data rights and infrastructure further highlights this issue. Best practices utilise Findable, Accessible, Interoperable and Reusable (FAIR) data principles.^36^ Additionally, the financing of personalised medicine efforts are increasingly trending towards outcomes-based models.^37^ These financial agreements require real world evidence collection to tie payments to results, which in turn requires robust and reliable data infrastructure.^37^ An ideal data infrastructure would enable transparent funding decisions in outcomes-based agreements and enable future research, while still protecting the data privacy and security rights of the individual.

Overall, there is a clear need for a standardised European framework for the value assessment of NGS/CGP diagnostics in oncology. Decision-making and uptake of NGS/CGP diagnostics in oncology remains considerably fragmented throughout Europe and even within nations.^8^ In Spain, for example, there are no standard procedures or nationally agreed guidelines for using NGS/CGP, leaving the decision-making to autonomous regions.^8^ This ultimately results in access inequities as individuals in wealthier areas benefit from greater access.^8^ The set of criteria and sub-criteria presented in this VAF serve as a starting point for healthcare systems seeking to implement a robust, validated and structured approach to the assessment of NGS/CGP diagnostic technologies. The simplest form of operationalising this framework is through a checklist approach, where evidence on a technology is synthesised for each value criteria, taking into account the resultant importance scores of each sub-criteria revealed in this framework. Another option, in line with the successful implementation of the IECS VAF, is the creation of stakeholder specific guidebooks and a free online course. More formally, the framework can be operationalised through a multi-criteria decision-making analysis (MCDA) approach, whereby specific weights are assigned to each sub-criteria based on preference elicitation in a local decision context.

Through this research, we propose a foundation for implementing a VAF for NGS/CGP diagnostics in oncology within the European context. There are few frameworks available that assess diagnostics and even fewer that assess NGS/CGP diagnostics in oncology. This research aims to contribute to the existing literature base by helping countries better approach their evaluation in a way that reflects real societal values and true utility of the technology. It is also the first NGS/CGP value framework to consider data usage and management after the test is performed. Moving forward, future pilots where HTA documents are written using it, as well as weighting exercises are needed to illustrate the value framework's practical application as a decision-making tool.

Some limitations were encountered in this study. Stakeholder subgroups were not the same size, ranging from three to eight participants, which may lead to a stronger influence of larger subgroups, and prevents the adequate performance of a subgroup analysis (see Supplemental Table S4). The scope of this study was limited to oncology and European stakeholders, which means this framework may not be immediately generalisable to other diagnostics, therapeutics and geographic areas. The framework could still be relevant to other settings though consideration of the scope for which it was created is necessary.

In conclusion, current HTA frameworks face challenges in accurately assessing NGS/CGP diagnostic technologies. The complexity arises from the link between diagnostic technologies and the benefits associated with multiple and ever-growing treatment options. This study identified key criteria highly valued by stakeholders, some of them not adequately considered in current HTA methods, such as data governance post-test. We propose a foundation for implementing a VAF for NGS/CGP diagnostics in the European oncology setting that has been co-created with relevant stakeholders. This research aims to address the gap in current VAFs for diagnostics and proposes value criteria that reflect real sentiments of key healthcare stakeholders.

## Contributors

Conceptualisation: Kanavos P, Augustovski F.

Data curation: Chavez D, Haig M, Main C,

Formal analysis: Chavez D, Haig M, Main C,

Funding acquisition: Kanavos P, Augustovski F, Mills M.

Investigation: Chavez D, Haig M, Main C,

Methodology: Kanavos P, Chavez D, Haig M, Main C, Mills M, Alcaraz A, Argento F, Colaci C, Alfie V, Pichon-Riviere A, Augustovski F.

Project administration: Kanavos P, Augustovski F, Mills M.

Supervision: Kanavos P, Augustovski F, Mills M.

Validation: Chavez D, Haig M, Main C.

Visualisation: Kanavos P, Mills M, Chavez D, Haig M, Main C.

Writing—original draft: Chavez D, Haig M, Main C.

Writing—review and editing: Kanavos P, Chavez D, Haig M, Main C, Mills M, Alcaraz A, Argento F, Colaci C, Alfie V, Pichon-Riviere A, Augustovski F.

All authors read and approved the final version of the manuscript. PK, FAu and MM have accessed and verified the underlying data.

## Data sharing statement

The data supporting the findings of this study are not publicly available. However, they are available from the corresponding author upon reasonable request.

## Declaration of interests

Authors have nothing to declare.

## Appendix A Supplementary data

Supplemental Material

Research Protocol
