# Supplementary material for: A Delphi study on valuing DNA sequencing in oncology: a European stakeholder developed framework for assessing next generation sequencing and comprehensive genomic profiling diagnostics
Source: eBioMedicine. 2025 Oct 16;121:105947. doi: 10.1016/j.ebiom.2025.105947 (PMC12554078; doi:10.1016/j.ebiom.2025.105947)
Supplement: Supplemental Material [file mmc1.docx]

# **Supplemental material**

# **Table s1. Criteria and sub-criteria with descriptions proposed to participants in rounds two to four**

| **Criteria** | **Sub-criteria** | **Description** |
| --- | --- | --- |
| Clinical impact | Clinical efficacy/effectiveness | The effect of NGS/CGP use and any resulting personalised medicine treatment decisions on patient clinical outcomes, including mortality, morbidity, and quality of life. |
|  | Test safety | Any adverse event(s) experienced by patients associated with the clinical procedures for test preparation, the performance of a test, or a change in therapeutic strategy due to the test results. |
|  | Consequences of wrong diagnosis | Clinical consequences of false positive/negative test results (e.g., delayed or foregone treatment due to missed diagnosis, or wrong treatment). |
| Test performance and quality | Test performance | The diagnostic yield and accuracy of the test (e.g., measures of sensitivity, specificity, precision and reproducibility), the number of results produced, and the amount of information generated. |
|  | Technical aspects | Technical characteristics of the test and the procedures that ensure accurate and reliable results, including library preparation, quality control, quality assurance and validation protocols. |
| Quality of scientific evidence | Quality of scientific evidence | The validity, credibility, and overall strength of the evidence used to support the decision to use NGS/CGP. |
| Non-clinical impact | Environmental impact | Impact of the test's production, use, implementation, disposal, and data storage and processing on the environment (e.g., the storage and processing of genetic sequences require computational memory and power, using significant energy resources). |
|  | Patient experience | Patients' perceptions, feelings, and interactions while undergoing the testing process through waiting for and receiving results, including treatment from staff (i.e., invasiveness, preparation and comfort). |
|  | Caregiver and/or family experience | The perceptions, feelings, interactions and burdens that caregivers and family members of the patient have throughout the testing process (e.g., coordination of appointments and logistics, emotional support, caregiving). |
|  | Impact on personal and family decisions | The impact of the diagnosis and treatment on one's personal decisions and that of their family, including education, career, lifestyle and family planning decisions or the need to test and make decisions on other family members for the possibility and consequences of sharing the mutation. |
|  | Cancer stigma | The impact that the diagnosis and subsequent treatment have at the personal level (e.g., exclusion, self-consciousness, rejection, blame by family and/or by society). |
|  | Bridge to other future treatments (¨Real option value¨) | Refers to the value of opportunity for patients to benefit from future R&D. To the extent that NGS/CGP improves therapeutic strategy and helps extend life, this creates opportunities for the patient to benefit from other future medical advances. |
| Impact on health system integration, organisation, and delivery of care | Impact on health service provision | Any infrastructural, technical, personnel, and patient care pathway modifications required to implement and provide testing (e.g., new specialised equipment, staff training, genetic counselling, and equipment maintenance) |
|  | Appropriateness of test use | The clinical, economic and/or non-clinical consequences of overuse, inappropriate use or abuse of NGS/CGP (e.g., overdiagnosis resulting in the provision of unnecessary treatments). |
| Economic aspects | Cost-effectiveness | A value-for-money comparison between costs and health outcomes of NGS/CGP to current practice or other diagnostic strategies. |
|  | Affordability | Budget impact for the payer when incorporating the test (e.g., acquisition costs, maintenance, supplies, training). |
|  | Financial impact on patients, carers or family | Out-of-pocket or indirect costs for patients, family, and caregivers associated with the testing process (e.g. caregiver time, transportation, and/or time off work for patients and their families). |
|  | Broader socioeconomic impact | Economic spillover effect on other sectors beyond health, including but not limited to, job creation/loss, data generation, industrial promotion, research and development processes, and technology transfer (a sector benefiting from innovations in another sector). |
| Ethical and governance concerns | Data security and privacy | The security and privacy of storage, processing, and dissemination of data produced by the test. |
|  | Informed consent and transparent communication | Voluntary, informed and explicit consent from the patient being tested regarding the collection, processing, analysis, storage, ownership, accessibility and sharing of personal data and test results prior to any sample collection. Communication to the patient about these issues is transparent and done in a way the patient can understand. |
|  | Data provenance | Test results are traceable and verifiable, meaning it is possible to follow the path of data through the different stages of development, manufacture, and distribution. |
|  | Ethical aspects | The relevant social norms that are used to evaluate challenges that arise from NGS/CGP (e.g., considerations around disclosure of incidental findings). It implies a consideration of the decision to implement or not implement NGS/CGP with respect to societal values versus the values constructed when NGS/CGP is in use. Note: this does not include the concept of informed consent, which has its own indicator. |
|  | Legal aspects | Refers to any legal or legislative considerations which may present barriers towards the provision of NGS/CGP. This may include but is not limited to, intellectual property, data protection regulations, medical liability, or health financing regulations. |
| Health system priorities | Disease burden | It is a measure of the total impact of the cancer on the population in terms of its prevalence, mortality, and morbidity (illness and disability or impact on health-related quality of life). |
|  | Disease severity | The extent or intensity of the cancer's impact on an individual's health. It is a measure of how serious or severe the disease is in terms of the impact on quality of life or disability, its duration, as well as its effect on mortality or life expectancy. |
|  | Unmet need | Tests targeting areas of high unmet need. |
|  | Research priorities | Priority for this technology and this disease in the current or future research agenda. |
|  | Equity | Impact of the test on equity and inequalities in the health system, including those concerning disadvantaged areas and populations, rare diseases and mutations, and catastrophic health expenditures, among others. |
|  | Public and population health | Any health benefits delivered at the population level from the implementation of NGS/CGP (e.g. through public health screening programmes or early detection programmes). |

#

# **Table s2. Demographic breakdown of participants who completed all four Delphi rounds.**

| **Stakeholder group** | **Number of participants** |
| --- | --- |
| HTA | 5 |
| Academia | 8 |
| Patient Advocacy | 6 |
| Physician | 4 |
| Industry | 4 |
| Decision-maker | 4 |
| Regulator | 3 |
| **Age** |  |
| 18-29 | 1 |
| 30-44 | 7 |
| 45-59 | 14 |
| 60+ | 11 |
| Undisclosed | 1 |
| **Gender** |  |
| Male | 22 |
| Female | 11 |
| Undisclosed | 1 |
| Country of Work |  |
| Spain | 5 |
| Germany | 6 |
| Italy | 4 |
| France | 2 |
| Denmark | 1 |
| Sweden | 2 |
| United Kingdom | 9 |
| Slovenia | 1 |
| Belgium | 1 |
| Netherlands | 1 |
| Switzerland | 1 |
| Austria | 1 |
| **Total** | **34** |

# **Table s3. Changes made to proposed sub-criteria in Round One**

| Sub-criteria | More information/description | Revised sub-criteria | Revised description | Merged with another sub-criteria | Deleted |
| --- | --- | --- | --- | --- | --- |
| Clinical consequences of test use | Clinical consequences during the diagnostic or therapeutic process of carrying out the test (e.g., a change in the therapeutic approach that is associated to an improvement in the state of health becomes apparent from the test result) | X | X |  |  |
| Test performance | Evaluates the diagnostic yield of the test through indicators such as sensitivity, specificity, precision and reproducibility |  | X |  |  |
| Technical aspects | Evaluates the stability and storage form of the reagents used to perform the tests. |  | X |  |  |
| Procedure safety | Unintended consequences in whom the test is being performed secondary to the performance of the test (e.g., injury to neighboring organs during biopsy) | X | X |  |  |
| Consequences of wrong diagnosis | Unintended consequences in whom the test is being performed secondary to the misdiagnosis (false positives and false negatives) |  | X |  |  |
| Safety of test preparation | Unintended consequences in whom the test is being performed secondary to the preparation for it (e.g., adverse events during bowel preparation for biopsy colonoscopy) |  |  |  | X |
| Safety for test operators | Unintended consequences in test operators (e.g. radiation exposure from taking tomography guided biopsies) |  |  |  | X |
| Risk of overutilization | Refers to the risk of overuse, or abuse, of genetic testing. (e.g: using the test in an individual, or a population, for reasons not specific to the patient's indication). | X | X |  |  |
| Quality of scientific evidence | How reliable the scientific evidence to be evaluated, as well as its results is; also the potential that different biases or systematic errors would not allow drawing valid conclusions. |  | X |  |  |
| Economic evaluation (Clinical effectiveness and/or Budget Impact Analysis) | Comparison between costs and health results of two or more diagnostic options. Budget impact for the funder when incorporating the test (e.g., includes acquisition costs, maintenance, supplies, training) | X | X |  |  |
| Other costs | Patient and family out-of-pocket expenses, costs related to productivity loss, etc | X | X |  |  |
| Impact on the health service provision system | Implementing the test requires modifications of buildings, processes, logistics, etc. within the organization providing health services |  | X |  |  |
| Impact on the patient care path | Implementing the test would be associated with less time in access to the benefit, additional studies, associated practices would be avoided, or the availability of resources would be increased |  | X | X |  |
| Health priority within the health system | Priority of this health problem (for the country or health system, defined by those who design health policies). |  |  |  | X |
| Research priorities | Priority for this technology and this disease in the current or future research agenda. |  |  |  |  |
| Disease burden | How important the loss of health, both in mortality and in quality of life is. This includes taking into account the pattern of inheritance, genetic heterogeneity, mutation prevalence, mutation penetrance and neomutation rate. |  | X | X |  |
| Test for neglected diseases | Test oriented to the diagnosis of neglected diseases |  |  |  | X |
| Test for communicable diseases and high prevalence | Test oriented to the diagnosis of communicable diseases and/or of high prevalence in the region (e.g. HPV/cervical cancer) |  |  | X |  |
| Test in populations with little access to health services | Test considered situations where there is poor access to health services |  |  | X |  |
| Test for disadvantaged or underserved Communities | Test oriented to the diagnosis of disadvantaged or underserved communities defined as those relevant communities that have been historically disadvantaged through discrimination, neglect, reduced research funding, or other factors. |  |  | X |  |
| Test for rare diseases | Test oriented to the diagnosis of rare neoplasic diseases. Definaded as a disease that affect a small number of people compared to the general population. In Europe, the European Medicines Agency (EMA) considers a disease with a prevalence of less than five in 10,000 people (equivalent to less than one in 2,000) to be rare. |  |  | X |  |
| Equity in health financing | Equity in health financing by promoting health, especially in disadvantaged areas, and helping to meet catastrophic health expenditures |  |  | X |  |
| Ethical and legal aspects | It considers relevant the social and moral norms and values that derive from the technology in question. It implies an understanding of the consequences of implementing or not to implement a sanitary technology in two aspects: with respect to the values that prevail in the society and with respect to the norms and values that the same technology constructs when it is put into use | X | X |  |  |
| Severity of the disease | It takes into account the risk of mortality, the risk of disability (and its severity), the quality of life, and the duration of type of cancer. | X | X |  |  |
| Absence of alternative diagnostic technologies | There is no diagnostic technology available for that type of cancer, or stage, etc. | X | X |  |  |
| Experience of who takes the test | Experience of who takes the test / caregivers (comfort, invasiveness, preparation) | X | X |  |  |
| Value of the information | Value of the information provided by the test in special situations (e.g., end-of-life diseases, diseases with poor prognosis, diseases that affect offspring) |  |  |  | X |
| Load on caregivers or family | The test is associated with a lower burden on caregivers or the family of whom the test is performed (e.g., the result of the test results in a lower number of subsequent controls or avoids other unnecessary tests or associated procedures) |  |  | X |  |
| Preparation and/or care | Pre-preparation and / or care after the test (characteristics, need for completion) |  |  |  | X |
| Number of results associated with the test | Number of results associated with the sample (amount of information provided by the test with the sample obtained |  |  |  | X |
| Test processing time | Sample processing time (suitable for the disease / target population) |  |  |  | X |
| Self-test | Self-test (whoever takes the test can do it himself or another person without the need for more training) |  |  |  | X |
| Fear of contagion | The early and correct diagnosis of an infectious diseases limits the spread the disease to others (e.g. HPV/cervical cancer). |  |  |  | X |
| Impact on education | The impact of the diagnosis and treatment on one’s education/schooling |  |  | X |  |
| Impact on career | The impact of the diagnosis and treatment on one’s career |  |  | X |  |
| Impact on stigma | The impact on the person or the family or society of the diagnosis or treatment, generating embarrassment, self-consciousness, rejection by family or rejection by society. | X | X |  |  |
| Environmental Impact | It is a measure that the production, use or implementation of technology would cause in the environment. Eg, technology is associated with a greater generation of toxic waste | X | X |  |  |
| Broader Social Impact | Impact on other sectors beyond health, such as job creation, industrial promotion, technology transfer, and society as a whole. | X | X |  |  |
| Innovation | The diagnostic test being evaluated uses new mechanisms or technologies that were not previously available or is a new test not known so far. |  |  |  | X |
| Quality assurance/ quality improvement program | Internal: What are the controls (positive or negative, normal or abnormal) and what are their origin (samples banking, cell lines)? Specify internal controls related to the analysis carried out and their limits. Are internal quality control procedures applied to the laboratory as a whole or specific to the test? Provide the rates of errors reported. Provide the type of standards (e.g., molecular weight markers) included in the analysis. External: Identify the external quality control programs for this test and specify the type of programs to which you participate. What does this program cover (e.g., analytical aspects of the test or interpretation and reporting)? If no external QC program exists, which type of control do you make (e.g., blind tests, exchanges of various samples between laboratories). |  |  | X |  |
| Public health/ population benefit | The public health/ population that this technology will bring when used for this health problem (e.g: detection of susceptible populations to improve their follow-up, screening uses) | X | X |  |  |
| Bridge to other future treatments  (¨Real option value¨) | When a health technology extends life, creates opportunities for the patient to benefit from other future advances in medicine. |  | X |  |  |
| External pressures | This driver assesses external pressures for test coverage, imposed by providers, members of patient societies, society at large, laboratories and politicians for accelerating unwarranted adoption of tests before solid evidence exists. |  |  |  | X |
| Degree of investment in research and development | The number of human subjects enrolled in the approval trials for the first indication, was used as a proxy for the research and development costs necessary to develop the drug. |  |  |  | X |
| Safety and data governance | This indicator refers to the extent to which test results and their associated patient data are protected from unauthorized access, use, loss, or corruption. | X | X |  |  |
| Effective access to the test and subsequent treatment | Real life effective coverage/access to the test and eventual treatment. |  |  |  | X |

# **Subgroup analysis**

A sub-group analysis of stakeholder groups was completed as an exploratory analysis, as the study size did not permit a robust subgroup analysis and therefore the results are not powered. Intra-rater agreement (IRA) assessed whether participants within each stakeholder group expressed similar judgements independently, enabling cross-group comparison. IRA was calculated using the Kappa statistic and Gwet’s agreement coefficient with linear weights, utilising a benchmark scale to assess levels of agreement. Wilcoxon’s test was calculated for each group to measure the stability of responses. Lastly, the non-parametric Kruskall-Wallis test was completed to understand whether any stakeholder groups disagreed with each other.

IRA of each stakeholder group showcases movement towards consensus within each group between rounds two, three and four (**Table s4**). However, no statistically significant results were found for patients due to a small sample size. All stakeholder groups had stable responses and there were no statistically significant disagreements between groups.

**Table s4**. Interrater agreement within stakeholder groups for criteria and sub-criteria in Delphi exercise

|  | Round 2 | | | | Round 3/4 | | | |
| --- | --- | --- | --- | --- | --- | --- | --- | --- |
|  | ***Ky*** | ***95% CI*** | | ***Benchmark Interval*** | ***Ky*** | ***95% CI*** | | ***Benchmark Interval*** |
|  | **Criteria** | | | | | | | |
| Academia | 0.50** | 0.25 | 0.74 | Moderate agreement | 0.55*** | 0.34 | 0.76 | **Substantial agreement** |
| HTA/regulators | 0.40** | 0.06 | 0.75 | Moderate agreement | 0.63*** | 0.42 | 0.84 | **Substantial agreement** |
| Decision Makers | 0.42** | 0.05 | 0.79 | Moderate agreement | 0.50** | 0.15 | 0.84 | Moderate agreement |
| Industry | 0.59*** | 0.23 | 0.96 | Moderate agreement | 0.54** | 0.22 | 0.85 | Moderate agreement |
| Patients | 0.29 | -0.18 | 0.77 |  | 0.3131 | -0.19 | 0.86 |  |
| Physicians | 0.67*** | 0.36 | 0.98 | **Substantial agreement** | 0.54** | 0.11 | 0.96 | Moderate agreement |
|  |  |  |  |  |  |  |  |  |
|  | **Sub-criteria** | | | | | | | |
| Academia | 0.33*** | 0.22 | 0.44 | Fair agreement | 0.45*** | 0.33 | 0.58 | Moderate agreement |
| HTA/regulators | 0.60*** | 0.52 | 0.68 | Moderate agreement | 0.74*** | 0.64 | 0.83 | **Substantial agreement** |
| Decision Makers | 0.22*** | 0.09 | 0.34 | Fair agreement | 0.41*** | 0.30 | 0.52 | Moderate agreement |
| Industry | 0.44*** | 0.30 | 0.59 | Moderate agreement | 0.63*** | 0.50 | 0.76 | **Substantial agreement** |
| Patients | 0.45*** | 0.34 | 0.56 | Moderate agreement | 0.67*** | 0.54 | 0.80 | **Substantial agreement** |
| Physicians | 0.49*** | 0.37 | 0.62 | Moderate agreement | 0.64*** | 0.52 | 0.76 | **Substantial agreement** |
|  |  |  |  |  |  |  |  |  |
|  |  |  |  |  |  |  |  |  |

Notes: Inter-rater agreement measured by the Gwet’s agreement coefficient with linear weights.

Benchmark scale of the level of agreement as suggested by Landis and Koch (1977): Coef. < 0.00 Poor agreement; 0.00 < Coef. ≤ 0.20 slight agreement; 0.20 < Coef. ≤ 0.40 Fair agreement; 0.40 < Coef. ≤ 0.60 Moderate agreement; 0.60 < Coef. ≤ 0.80 Substantial agreement; 0.80 < Coef. ≤ 1 Almost perfect agreement [1].

*p<0.1; **p<0.05; ***p<0.01

Source: The authors from analysis of Delphi data.
