# Supplementary material for: A Delphi study on valuing DNA sequencing in oncology: a European stakeholder developed framework for assessing next generation sequencing and comprehensive genomic profiling diagnostics
Source: eBioMedicine. 2025 Oct 16;121:105947. doi: 10.1016/j.ebiom.2025.105947 (PMC12554078; doi:10.1016/j.ebiom.2025.105947)
Supplement: Research Protocol [file mmc2.docx]

**Research Protocol**

The methodology for this project consists of four main stages:

1. Conducting a literature review to adapt an existing value framework.
2. Recruiting relevant stakeholders for input and feedback.
3. Adapting the framework based on stakeholder contributions.
4. Performing a statistical analysis of the collected data.

Additionally, the protocol includes a **Data Management** section, which outlines the procedures for handling and protecting data throughout the project.

**Process diagram**

**Stage 1: Literature Review and Initial Value Criteria and Value Sub-criteria Identification**

- The **Diagnostic Test Value Framework for Latin America** had previously identified 20 existing value frameworks, which were used as a foundation for the updates and changes made in the current review.
- A **systematic review (SR)** was conducted to identify value criteria and value sub-criteria that could potentially modify and adapt the existing value framework. The SR included a comprehensive search of both grey literature and primary research on relevant frameworks.
- The identified value criteria and sub-criteria were **mapped onto the existing framework**, ensuring that it accounted for the differential aspects of NGS-CGP (Next-Generation Sequencing-Comparative Genomic Profiling) technology.
- The **outcome** of this stage was a set of initial value criteria and value sub-criteria that served as the basis for the initial round of stakeholder engagement.
- The systematic review conducted in the first stage of the project was not registered, so no registration number is available. However, it was rigorously reported in accordance with the PRISMA checklist.

**Stage 2: Stakeholder Recruitment**

Two groups will be formed: a **steering committee (SC)** and a wider **collaborative stakeholder group**. Both the steering committee and the collaborative stakeholder group will be invited to participate in the framework adaptation activities described in Stage 3 through formal invitation letters.

Participants in both the steering committee and the collaborative stakeholder group will be identified by researchers through established professional networks. These networks included prominent organizations such as the ADVANCE HTA consortium, the IMPACT HTA consortium, WHO Europe Collaborating Centres, health insurance and payer organizations, HTA agencies, regulatory bodies, professional societies (e.g., ESMO), and patient advocacy groups, among others.

- The **steering committee** will established to oversee the research process and provide high-level guidance. As stated in the invitation to prospective members, the SC was tasked with advising on project methodology, overseeing IECS-LSE activities, and contributing to stakeholder group discussions. The SC members' expertise in value assessment and funding decisions for health technologies was considered essential for ensuring the research's validity and long-term sustainability.

Steering committee members will be invited via a formal letter and were expected to:

- - Participate in **four 60–90-minute meetings** over 12 to 18 months, including a kick-off meeting and one meeting at the end of each six-month research phase.
  - Advise on **project methodology and approach**.
  - **Review and approve** project deliverables.
  - Participate in **two or three rounds of stakeholder group preference elicitation** and consensus generation for adapting the VAF (Value Assessment Framework).

**Steering Committee Members:**

- Panos Kanavos – London School of Economics
- Federico Augustovski – Institute for Clinical Effectiveness and Health Policy
- Benjamin Horbach (Global Leader, Market Access, Roche, representing PCC)
- Jean Mossman – Patient advocacy
- Mark Lawler – Translational Cancer Genomics / Queen´s University Belfast
- Michael Drummond - Professor of Health Economics / University of York
- Nicola Normanno - Italian Cancer Society
- Laura Sampietro-Colom - Health Technology Assessment / Hospital Clinic Barcelona
- Albrecht Stenzinger - Institute of Pathology, University Hospital Heidelberg, Germany
- The **collaborative stakeholder group** will be formed by a minimum of 30 participants from European countries (focusing on the EU5 countries, as well as Sweden, the Netherlands, Austria, and Belgium). Participants will be divided into six subgroups based on their roles within the healthcare system. Similar numbers of participants will be recruited from each group to ensure balanced representation. All participants were required to have worked or be currently working in one of the study countries and represent one of the following categories:
  - Patient advocacy /civil society (e.g., ECPC-European Cancer Patient Coalition, Patvocates Research)
  - Decision-makers (e.g., MoH, EU, Procurement, HTAs, Hospital decision-makers)
  - HTA environment/ Regulators (e.g., EMA or notified bodies)
  - Academia (e.g., Bocconi University, Stockholm School of Economics, University of Reims)
  - Industry (e.g., Marivek Healthcare Consulting, MedTech)
  - Physicians (e.g., Italian Cancer Society, Comprehensive Cancer Centre)

Identified individuals will be invited via email, with a formal letter outlining the project's objectives, their expected roles, participation details, and compensation. The invitations will be managed centrally by a single researcher, who is responsible for contacting potential participants via email while copying research managers on the correspondence to ensure proper oversight.

In addition, participants will be allowed to **suggest additional potential members** for the panel. These suggestions will be reviewed by the research team to ensure that new panellists met the necessary criteria and helped maintain balance and representation across stakeholder groups.

Participants will be compensated **€500** for completing the study, while steering committee members will receive additional compensation based on their broader scope of involvement.

**Stage 3: Framework Adaptation**

The adaptation process will begin with the **starting framework**, adapted from the IECS Value Assessment Framework (VAF) for in-vitro diagnostics in Latin America (LATAM). This adaptation is based on findings from a systematic literature review (see Stage 1 for details). The framework adaptation process was submitted to the **LSE Ethics Committee** for approval prior to the start of the Delphi process.

Before the official launch, the **web platform** and the initial framework presentation will be **piloted** with researchers from the group who were not directly involved in managing or uploading the process. The pilot serves to ensure the platform functions as intended and that framework and the instructions are clearly understood by participants. Feedback from this internal pilot will help refine both the presentation of the framework and the structure of the online platform before wider stakeholder engagement begins.

The Delphi exercise involves conducting four rounds using Welphi, an online survey platform designed specifically to implement the Delphi method. Welphi creates questionnaires to gather opinions from geographically dispersed participants, ensuring anonymity and promoting consensus through a non-face-to-face format.

Participants who do not complete a Delphi round will be excluded from the following round. For example, participants who fail to complete Round 1 are not invited to Round 2. Calendar invites and reminders are to be sent to participants at the beginning of each round to ensure engagement and timely completion.

**Step 1: Delphi Round 1 - Open-Ended Feedback**

The goal of the first round is to present the IECS-adapted framework to participants and obtain feedback in the context of NGS/CGP diagnostics in Europe.

1. **Background Information Presentation**
   - Participants will be introduced to the background and scope of the project: NGS/CGP technologies:

Current impact and future expectations on the health system. NGS/CGP for diagnostics in Europe: The role and integration of these technologies.

1. **Framework Presentation**
   - Participants will be shown the value framework separated into value criteria, including detailed explanations of each.
2. **Feedback Collection**
   - Instructions for R1 are as follows:

*Please review each of the following value statements and proceed as follows: If you think there are issues with the wording or content of a statement, please provide your feedback using the comment button to the right of the value statement.*

*If you believe that we are missing any aspects of value regarding NGS/CGP technology assessment, please click on the ‘add sub-criteria’ button at the bottom of the page and write your thoughts. Try to explain your suggestion as specifically and objectively as possible.*

*Please keep in mind that the purpose of this framework will be to inform decision makers at the time of making coverage, reimbursement or funding decisions.*

- - Participants will be invited to provide comments, suggest additional criteria, and adjust value criteria and sub-criteria where necessary.
  - For each new sub-criteria proposed, participants will be asked to provide an explanation, assign it to an existing value criterion, or suggest a new criteria.

1. **Context-Specific Feedback**
   - Open-ended questions will be posed about NGS/CGP for oncology and rare diseases, asking whether certain sub-criteria were relevant only to these diagnostic contexts.
   - Instructions and the questionnaire for the open-ended section of R1 are as follows:

*Open Page*

- *In your opinion, how should these criteria be grouped?*
- *Write at least three criteria in which the criteria can be grouped.*
- *For example, one criteria could be “clinical characteristics”.*

1. **Round Duration**
   - This round of the Delphi process will remain open for 10-14 days to allow sufficient time for participants to contribute.

**Step 2: Thematic Analysis**

The purpose of this step is to systematically review the feedback received and present a revised framework incorporating participant views.

1. **Data Importation**
   - All comments and proposed criteria from the participants will be imported into Microsoft Excel for analysis.
2. **Coding and Analysis**
   - The data will be coded by theme and value criteria, ensuring all comments are categorized appropriately. This process will be carried out by three researchers, with two researchers reviewing and coding each case. Any disagreements will be resolved by the third researcher, who acted as an arbitrator to ensure consistency and accuracy in the analysis.
3. **Incorporation of Feedback**
   - **Meetings will be conducted** to extensively review participant comments and feedback from Round 1. During these sessions, participant feedback will be incorporated into the appropriate sub-criteria. Duplicates or previously addressed proposals will be removed during the review process, and newly proposed sub-criteria will be reviewed by theme. Where necessary, sub-criteria will be merged or reworded for clarity and consistency to ensure a well-organized and coherent framework.
4. **Revised Framework**
   - A final list of value sub-criteria, including revised, new, and unchanged elements, will be produced and organized by value criteria.
   - A side-by-side comparison of the initilly presented framework and the revised framework will be conducted to highlight the changes.
5. **Timeframe**
   - This stage will be completed over approximately in one month.

**Step 3: Delphi Round 2 - First Value Judgements**

This round aims to collect participants' value judgements on the importance of each value criteria and value sub-criteria for assessing the value of NGS/CGP diagnostics in Europe.

1. **Value Judgement Process**
   - Participants first will rate each **value criteria** on a **Likert scale** ranging from 1 to 5, where 1 represented "not at all important," 2 "low importance," 3 "moderate importance," 4 "important," and 5 "very important." Following this, value sub-criteria will be presented in **separate sections** corresponding to each value criteria on the web platform, and participants will then be asked to assess each sub-criteria using the same scale.
   - Instruction for this round are as follows:

Value Criteria:

*REQUIRED: Independently score the importance level of each criteria on an ‘importance’ Likert scale, ranging from ‘1: Not at all important’ to ‘5: Very important’.*

*OPTIONAL: You may provide a comment if any aspects are unclear. Please note comments will be used for research context only and will not be used to change sub-criteria for the next round.*

Value subcriteria:

*REQUIRED: Please review each of the following value statements in 8 criteria (Clinical impact; Test performance and quality; Quality of scientific evidence; Non-clinical impact; Impact on health system integration; organisation; and delivery of care; Economic aspects; Ethical, legal and data governance aspects; and Health system priorities) according to their importance within a framework for assessing NGS/CGP technologies for oncology. Please score each statement according to a 5-point importance Likert scale, ranging from ‘1: not at all important’ to ‘5: very important’. Please keep in mind that the purpose of this framework is to act as a decision aid for reimbursement and coverage decisions.*

*OPTIONAL: Please comment on the associated sub-criteria if its wording and/or concepts are unclear. Note: comments will be used for research purposes only and will not impact changes in the framework for the next round.*

1. **Mandatory Judgements**
   - Participants will be required to provide a value judgement for each value criteria and sub-criteria.
2. **Round Duration**
   - This stage of the Delphi process will be kept open for 10-14 days.

**Step 4: Delphi Round 3 - Second Value Judgements**

The purpose of this round is to provide participants with the opportunity to revise their previous responses after seeing the collective feedback from Round 2.

1. **Presentation of Results**
   - Participants will be shown the dispersion of responses for each value criteria and value sub-criteria from Round 2, along with their own prior responses.
2. **Opportunity to Revise**
   - Based on this information, participants will be allowed to revise their previous ratings or leave them unchanged.
   - The directions for this section will be similar to those provided in Round 2, with slight adjustments where necessary.
3. **Round Duration**
   - This stage of the Delphi process will remain open for 10-14 days.

**Step 5: Delphi Round 4 - Final Value Judgements**

The purpose of the final round is to stabilize the participants’ judgements, allowing for confirmation and validation of their responses, leading to more robust results.

1. **Validation Process**
   - Similar to Round 3, participants will be shown the distribution of responses and their previous answers. They will be asked to confirm or revise their ratings for each value criteria and sub-criteria.
   - The directions for this section will be similar to those provided in Round 2, with slight adjustments where necessary.
2. **Final Stability**
   - This final round aims to establish greater stability in the results, ensuring that the findings reflected the consensus reached through multiple stages of feedback.

**Stage 4: Statistical Analysis Using Stata**

The objective of this stage is to evaluate the level of agreement across stakeholder groups regarding the importance of NGS/CGP diagnostics in Europe. A series of statistical tests will be applied to analyze participant consistency, agreement, and to identify any significant disagreements between groups. The analyses to be conducted include:

1. **Interrater Agreement (IRA)**: The **Kappa statistic** and **Gwet’s Agreement Coefficient** is employed to measure the extent to which individuals within each stakeholder and country group independently made similar judgments. These metrics assess internal agreement within groups, and groups demonstrating a high degree of internal consistency were compared against one another.
2. **Wilcoxon Test**: This test is used to evaluate the stability of responses within stakeholder groups across Delphi rounds. When significant instability is detected for certain sub-criteria, additional Delphi rounds are considered to achieve consensus.
3. **Interquartile Range (IQR)**: The IQR of the medians is calculated to determine the level of agreement across stakeholder groups. This metric highlights consensus or divergence regarding the importance of specific sub-criteria.
4. **Kruskal-Wallis H Test**: This non-parametric test is used for subgroup analysis to determine whether there are significant disagreements between stakeholder groups on each sub-criteria. Where significant differences are found, **Dunn’s test** is conducted to identify which specific groups disagreed.

Statistical tests will be completed using **STATA 16.1 software** to assess consensus, stability, and median responses across the cohort. Consensus will be evaluated using the **Interquartile Range (IQR)**, with consensus defined as an IQR ≤ 1. The **Wilcoxon test** will be applied to assess the stability of responses between rounds 2 and 3, and between rounds 3 and 4, to determine whether participants changed their opinions over time.

The **inclusion criteria** for the final framework considers both the IQR (≤ 1) and the median response for each sub-criteria. Sub-criteria with a median of "important" or "very important" are classified as **essential** for value assessment, while those with a median of "moderately important" or lower are classified as **complementary** to the assessment process.

**Data Management and GDPR Compliance**

This study will adhere to the General Data Protection Regulation (GDPR) to ensure the privacy and protection of personal data. All personal data collected during the course of this study will be processed and managed in accordance with GDPR guidelines, ensuring the following principles are upheld:

- Lawful, Fair, and Transparent Processing: Personal data will be collected and processed for specified and legitimate purposes. Participants will be fully informed about the nature and scope of the data collection and its intended use.
- Data Minimization: Only data that is strictly necessary for the research purposes will be collected and retained.
- Consent: In compliance with GDPR, informed consent will be obtained from all participants prior to data collection. Participants will be informed of their rights, including the right to withdraw from the study at any time without any adverse consequences.
- Data Security: Appropriate measures will be taken to safeguard all personal data, including secure storage, and controlled access.
- Anonymization: Where possible, data will be anonymized to protect the identities of participants. Identifiable information will be separated from research data, and all analysis will be conducted on de-identified datasets.
- Data Breach Policy: In the event of any data breach, participants will be promptly notified, and the relevant authorities will be informed, in accordance with GDPR's breach notification requirements.

This study will seek ethics approval, and all data handling protocols will be reviewed and deemed compliant with GDPR requirements.
